# A Novel Tube Thoracostomy Simulation Model for the Deployed or Resource-Limited Environment

**DOI:** 10.7759/cureus.8901

**Published:** 2020-06-29

**Authors:** Ryan Walsh, Scott Young, Zachary Sletten

**Affiliations:** 1 Emergency Medicine, Vanderbilt University Medical Center, Nashville, USA; 2 Emergency Medicine, Madigan Army Medical Center, Tacoma, USA; 3 Emergency Medicine, San Antonio Military Medical Center, San Antonio, USA

**Keywords:** tube thoracostomy, medical simulation, austere, military, trainer, simulation trainer, chest tube

## Abstract

Tube thoracostomy placement is a critical, life-saving procedure often performed in the setting of trauma for the treatment of intrathoracic injury, such as hemothorax or pneumothorax. It also represents a high-acuity low-occurrence (HALO) event for many medical providers who may have limited opportunities for practice and mastery, particularly those in austere or resource-limited environments, such as in the deployed setting. Simulation offers opportunities for practicing the techniques required to properly perform and master the procedure. Finding affordable and accessible models for training, however, still represents a significant obstacle for many medical providers. We present a novel tube thoracostomy simulation model assembled from low cost and readily available materials.

## Introduction

Tube thoracostomy placement is a critical skill, often performed in an emergent setting for the accumulation of air or fluid in the thoracic cavity [1]. When performed for the emergent evacuation of the pleural space, it can be life-saving [2]. This procedure is often considered for thoracic trauma, with thoracic injuries reported in 12%-50% of civilian trauma and 10% of combat-related trauma [3]. It is a skill integral to advanced trauma life support and is among the skills on the Individual Critical Task List (ICTL) for which military physicians are required to maintain proficiency to ensure deployment readiness [4,5]. This is a high-stakes, high-stress procedure given that proper and timely performance of the procedure is critical not only for its potential to treat life-threatening injuries, but also because it is associated with a variety of complications, such as infection and injuries to the heart or major vessels, the lung, diaphragm, nerves and abdominal organs. Moreover, delays in performance can be associated with increased morbidity and mortality [2]. Overall, complications are associated with 5%-10% of tube thoracostomies [6]. Training has been demonstrated as a means of reducing these complications, and more specifically simulation-based training has demonstrated the ability to improve performance and confidence for those with limited experience in trauma [5,7]. Currently, there are a variety of methods to teach and practice this procedure to include cadavers, live tissue training and manikins, which can be costly, require regular maintenance and may offer limited opportunities for practice [7]. We discuss a tube thoracostomy trainer which can be assembled with inexpensive and readily available materials and offers opportunities for continued practice and training in austere and remote environments. 

## Technical report

**Figure 1:**
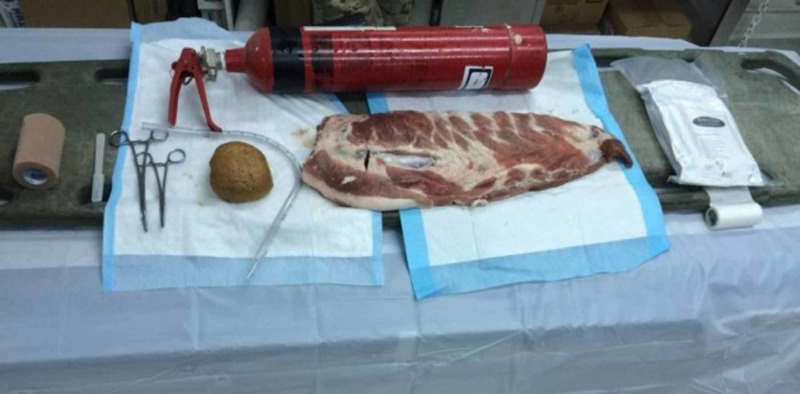
Equipment for tube thoracostomy model

In a military deployed environment with limited personnel, supplies and equipment, we created a simple tube thoracostomy insertion model. The model was made of readily available materials found in our role II environment (limited medical and surgical capabilities) modifying a previously described task trainer (Figure 1) [8].

**Figure 2:**
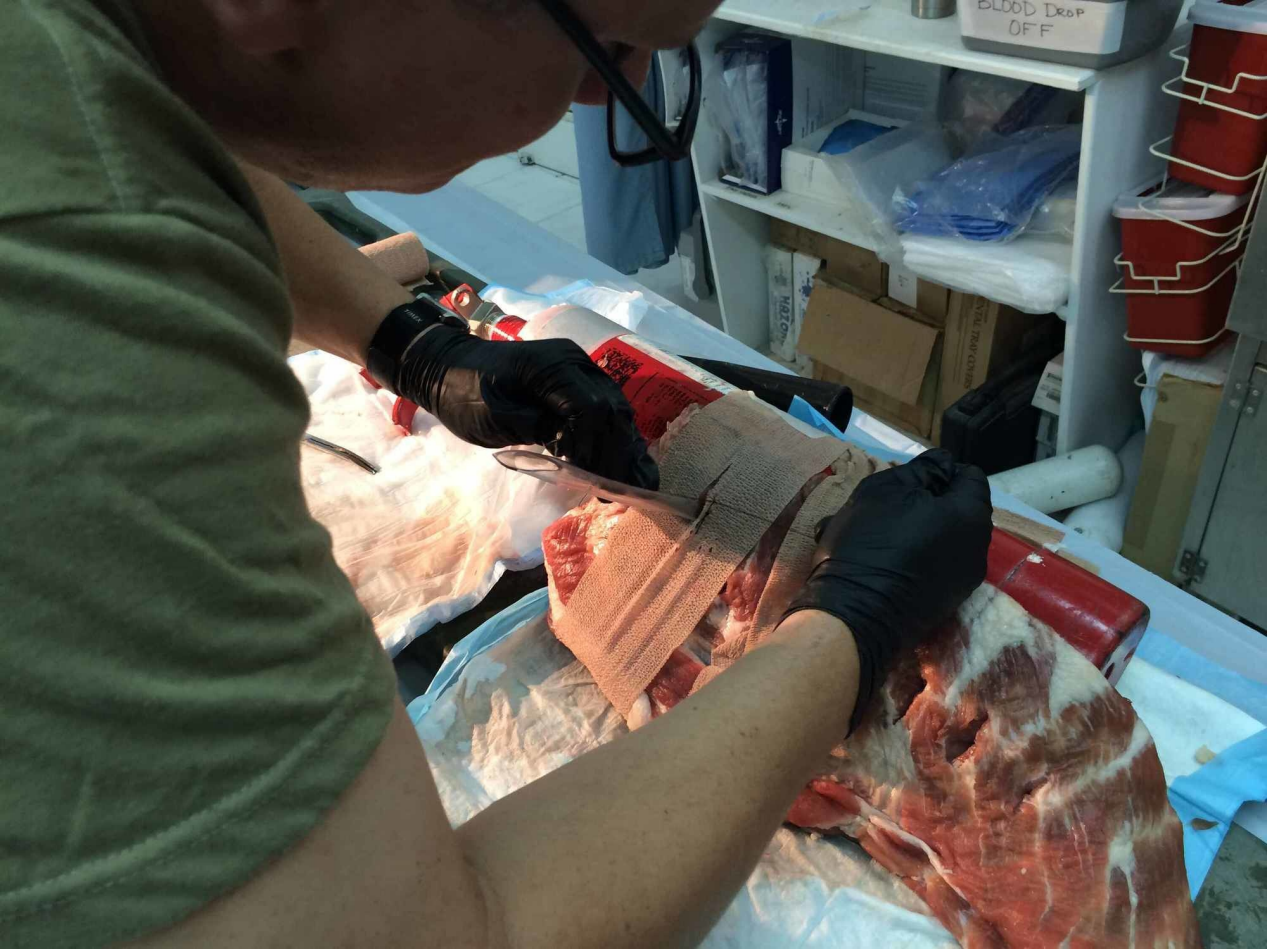
Inserting chest tube into model

A large rack of bovine ribs was secured with self-adherent wrap, representing skin, to a size two fire extinguisher. The fire extinguisher had the benefit of providing stability to the model as well as representing the mediastinal structures when advancing the chest tube. A partially inflated nitrile glove was placed behind the ribs to simulate a collapsed lung while a bagel was placed inferior to the glove to simulate the diaphragm. The glove and bagel were able to be palpated digitally by the participant to confirm correct location of the incision prior to inserting the tube. A deployable field chest tube kit from Bound Tree Medical (Dublin, OH) was used to perform the tube thoracostomy procedure in standard fashion (Figure 2) [2].

The model was able to be reset in less than five minutes by repositioning the rack of ribs on the fire extinguisher as well as repositioning the self-adhering wrap up or down a rib space, allowing all participants to train on one rack of ribs in an efficient manner.

A total of 17 students participated, 13 of whom had limited to no experience with inserting a chest tube. Participants included one orthopedic surgeon, two family practice physicians, one certified registered nurse anesthetist (CRNA), two intensive care unit (ICU) nurses, five licensed practical nurses (LPNs) and six combat medics. Prior to hands-on training, participants received 30 minutes of didactic instruction from an emergency medicine physician on how to properly insert a chest tube. Participants were then required to demonstrate proficiency in tube thoracostomy insertion by ensuring proper standard procedural steps were followed.

All participants were able to show proficiency in tube thoracostomy placement and reported increased confidence in their ability to perform the procedure despite 76% (13/17) of participants having limited to no prior experience in chest tube insertion. Of the four participants who had prior experience with chest tube insertion, all agreed the model had high fidelity. One physician stated that training on the model increased his skill to the point where he was able to confidently place a chest tube on his own in the deployed environment without supervision.

## Discussion

Tube thoracostomy placement is part of the advanced trauma life support curriculum and a critical task in the management of thoracic trauma. Effective training in this skill is essential for medical providers around the world, irrespective of available resources. We created a low cost, reproducible chest tube insertion model using commonly found items in many resource-limited environments. This simulator can be easily constructed and deconstructed in a short amount of time with minimal supplies. Several of the items described in our original model could easily be substituted with other objects depending on availability. Food elastic wrap, foam tape, or another stretchable material could replace the self-adherent wrap as the “skin." Any dome-shaped object could replace the bagel. The fire extinguisher acts as an anchor for the model; any type b oxygen cylinder or other elongated weighted cylinder would easily suffice.

Creating a high-fidelity yet cost-effective simulation model can be challenging for invasive medical procedures. Fidelity in medical simulation is defined, in this case, as the extent to which a simulator represents the experience of performing a tube thoracostomy [9]. Although we feel our model offers high fidelity, previous literature has demonstrated that high-fidelity models may not have a significant advantage over low-fidelity models when it comes to transfer of learning [10]. Our improvised tube thoracostomy simulator was well received by the group and resulted in improved proficiency in procedural performance.

Commonly used tube thoracostomy simulators can cost between 500 and 2,000 United States dollars (USD) or more. The non-reusable aspects of our model may cost roughly 15 to 20 USD per training session. Most of this cost is the "rack of ribs." More than 130 simulation sessions could be held using our proposed model for the cost of a proprietary simulator. Additionally, the improvised simulator would not have the same storage and maintenance requirements common to proprietary models. 

The diverse group that participated and benefited from this training demonstrates the value this model may have on providers at different levels of medical training. From board-certified surgeons to combat medics, most participants felt like they were better prepared to perform this life-saving intervention.

The tube thoracostomy training occurred as an isolated event. Integration into a full simulation scenario may change the user's perception of its effectiveness. The performance of a complicated medical procedure on any simulation device may lead to a false sense of confidence on the part of the user, especially when performed in isolation. The number of participants in this training was relatively low. Further study on a larger and more diverse group could provide more evidence to the effectiveness of this low-cost improvised tube thoracostomy simulator.

## Conclusions

Tube thoracostomy continues to have an essential role in the treatment of significant thoracic trauma. Our tube thoracostomy training model can be created using supplies commonly available in limited-resource environments. This task trainer can be used to increase the skill level and proficiency of medical providers from many different disciplines. 
